# Electrochemically Modulated Optical Imaging Sensors Integrated with Microfluidics

**DOI:** 10.3390/bios16020086

**Published:** 2026-01-30

**Authors:** Zehao Ye, Jiying Xu, Yi Chen, Pengfei Zhang

**Affiliations:** 1 Beijing National Laboratory for Molecular Sciences, Key Laboratory of Analytical Chemistry for Living Biosystems, Institute of Chemistry, Chinese Academy of Sciences, Beijing 100190, China; 2 University of Chinese Academy of Sciences, Beijing 100049, China

**Keywords:** microfluidic, surface plasmon resonance, plasmonic scattering imaging, electrochemical sensing

## Abstract

Microfluidics has emerged as a powerful platform for the analysis of minute sample volumes, driving its widespread adoption in biosensing applications. Optical imaging and electrochemical sensing are two typical integration strategies, each offering distinct advantages. The optical methods provide detailed spatial mapping of chemical processes, while electrochemical techniques enable selective detection that is unhindered by optical scattering from impurities. Here, we introduce a novel optical imaging–electrochemical sensor for integrated microfluidic analysis. This approach employs an electrochemical workstation to modulate optical signals, enabling the simultaneous acquisition of decoupled optical images and electrochemical readings. Consequently, it delivers complementary information, revealing both the spatial distribution of analytes and their intrinsic electrochemical properties. We detail the system design and imaging principle, demonstrate its utility through the analysis of noble metal nanoparticles, which are commonly used for signal amplification in biosensors, and finally apply it to monitor biological processes on live cells. We believe this integrated methodology will develop into a powerful tool for operando analysis in microfluidics, significantly expanding its application in the biosensing of complex biological fluids.

## 1. Introduction

The integration of multiple analytical modalities within microfluidic systems is essential for comprehensive biochemical analysis, yet creating truly synergistic, high fidelity sensor platforms remains a formidable challenge [1,2,3]. Microfluidics provides unparalleled control over minute sample volumes, fueling advancements in chemical sensing, point of care diagnostics, and single cell analysis [3,4,5]. To fully exploit this potential, complementary detection methods are required. Optical imaging delivers non-invasive, spatially resolved dynamic information, while electrochemical sensing offers quantitative sensitivity and selectivity that are unimpeded by optical scattering [6]. However, simply co-localizing these techniques fails to capture their synergistic potential; a truly integrated platform where one modality directly and precisely modulates the other is needed to unlock operando insights into complex interfacial processes.

Electrochemical surface plasmon resonance (EC-SPR) imaging represents a significant step toward this goal, merging the surface-sensitive optical detection of SPR with electrochemical control [7,8,9,10,11,12,13,14,15,16,17,18,19,20]. This allows for the optical readout of faradaic currents and impedance, correlating electrochemical activity with spatial information. Despite its promise, conventional EC-SPR is fundamentally limited by the physics of propagating surface plasmons. The diffraction-limited, long-range propagation of plasmon waves produces blurred images with a tail of larger than 10 µm, resulting in poor spatial resolution, obscuring heterogeneous analysis at the nanoscale. Furthermore, the SPR signal is highly susceptible to background from surface charging, which can swamp the optical signature of low-abundance analytes, severely limiting sensitivity and quantitative accuracy in complex media.

Here, we introduce a microfluidic imaging sensor that overcomes these barriers by leveraging plasmonic scattering microscopy (PSM) [21,22,23,24,25,26]. We engineer a platform where electrochemical modulation is coupled not to the intensity of reflected plasmonic waves, but to the scattered light from local dielectric perturbations. This scattering-based paradigm inherently suppresses long-range propagation artifacts, yielding a spatial resolution that is nearly an order of magnitude superior to standard SPR imaging. Crucially, the PSM signal demonstrates remarkable insensitivity to surface charging, effectively isolating the optical response to specific faradaic events and drastically improving the signal-to-background ratio (Supplementary Note S1). We detail the design and operating principle of this integrated opto-electrochemical microscope. We first demonstrate its capability by resolving the distinct cyclic voltametric profiles of individual noble metal nanoparticles, highlighting its superior spatial resolution for electrocatalytic studies. We then deploy the platform to monitor biological processes in live cells under microfluidic perfusion, showcasing its sensitivity for label-free biosensing.

## 2. Materials and Methods

### 2.1. Materials

Gold nanocubes (AuNCs) with a side length of 90 ± 5 nm were purchased from NanoSeedz (Woburn, MA, USA). Silver nanowires with a diameter of 68 ± 8 nm and a length of 11.8–13.4 μm were obtained from NanoSeedz (Woburn, MA, USA). Dopamine hydrochloride (Cat. No. A11136.06) was purchased from ThermoFisher Scientific (Waltham, MA, USA). Tris-HCl (Cat. No. T6044) was purchased from Macklin Biochemical Co., Ltd. (Shanghai, China). Potassium thiocyanate (KSCN, Cat. No. P3011) was purchased from Sigma-Aldrich (St. Louis, MO, USA). Histamine dihydrochloride (Cat. No. H110868) was purchased from Aladdin Biochemical Technology Co., Ltd. (Shanghai, China). Attachment factor solution (Cat. No. S-006-100), penicillin–streptomycin mixture (Cat. No. 15140122), fetal bovine serum (Cat. No. 10437036), Trypsin-EDTA (0.05%, Cat. No. 25300120), and live cell imaging solution (Cat. No. A14291DJ) were purchased from ThermoFisher Scientific (Waltham, MA, USA). The fetal bovine serum was inactivated by heating to 56 °C for 30 min. Eagle’s Minimum Essential Medium (EMEM, Cat. No. 30-2003), and Kaighn’s Modification of Ham’s F-12 Medium (F-12K, Cat. No. 30-2004) were purchased from ATCC (Manassas, VA, USA). A 25-cm^2^ flask (Cat. No. 3289) was purchased from Corning Inc. (Corning, NY, USA). A 0.9-cm^2^ flexi-perm silicon chamber was purchased from Sarstedt AG & Co. KG (Nümbrecht, Germany). The BK7 cover glass (Cat. No. 48366067) was purchased from VWR International LLC (Radnor, PA, USA). Immersion oil (Cat. No. 16482) was purchased from Cargile Laboratories (Port Jefferson, NY, USA).

### 2.2. Cell Culture

The A549 (Cat. No. CCL-185) and HeLa (Cat. No. CCL-2) cells were purchased from ATCC (Manassas, VA, USA). All the cells were cultured in a 25-cm^2^ flask at 37 °C with 5% CO_2_ and 70% relative humidity. The A549 cells were grown in F-12K with 10% fetal bovine serum and 1% penicillin–streptomycin mixture. The HeLa cells were grown in EMEM with 10% fetal bovine serum and 1% penicillin–streptomycin mixture. Cells were passaged with 0.05% Trypsin-EDTA when they were approximately 80% confluent.

### 2.3. Surface Functionalization

For AuNC and silver nanowires analysis, gold-coated glass slides, which were fabricated by coating BK7 glass cover slides with 2 nm of Cr, followed by 48 nm of gold via a vacuum evaporator (JEE-420, JEOL, Tokyo, Japan), were used and first modified by 5 mg/mL dopamine in the Tris-HCl buffer (10 mM, pH 8.5) in the dark for half an hour, and then washed with electrolyte and ready for instant introduction of AuNC or silver nanowires for analysis. For cell analysis, before attaching the cells to the sensor surface, ~600 μL attachment factor solution was added to the flexi-perm silicon chamber on the gold-coated glass slide, and then incubated in the incubator at 37 °C. Then, the attachment factor solution was removed, and the gold coated glass slides were dried naturally in the bio-hood. After drying for ~8 h, ~20000 cells were added to each flexi-perm silicon chamber.

### 2.4. Data Processing

The raw PSM images were recorded at a frame rate of 100 frames per second. Postprocessing of data was accomplished using programs written in Matlab (Version R2023a). For the achievement of cyclic voltammetry (CV) curves, the intensity variation curve of each pixel or region of interest (ROI) of the raw PSM images was first denoised by polynomial fitting and then converted into a CV curve by deconvolution calculation [27]. For the AuNC, the TrackMate plugin (Version 7.1.1) in ImageJ (Version 1.54r) was employed to find and count particles, and the PSM image intensity variation curve of a particle was determined by integrating the intensities of all pixels within the Airy disk. For the silvernanowires, the ROI manager of ImageJ was employed to select the area of singlenanowires, and the Plot Z-axis Profile plugin was used to achieve the PSM image intensity variation curve. From the CV curves, the oxidation/reduction peak currents and their corresponding potentials and the full-widths at half-maximum (FWHMs) could be extracted. For the achievement of electrochemical impedance, a fast Fourier transform (FFT) analysis was performed on each pixel of the PSM image sequence, with a sampling time of 5 s and a rolling step of 1 s. So, 500 raw PSM images were processed pixel-by-pixel by FFT to create an amplitude image at the modulation frequency of 28 Hz to create an electrochemical PSM (EC-PSM) image and averaged to create a PSM image. The cell boundaries for recognizing single cell adhesion regions were precisely localized, and individual cell adhesion regions were further subdivided into four geometrically uniform ROIs. Then, the mean intensity within the single cell adhesion regions or ROIs was used to determine the whole cell or ROI signal.

### 2.5. Experimental Conditions

During the nanoparticle experiments, semi-open square microfluidic channels with a side length of 10 mm and height of 1 mm were adopted (Supplementary Note S2). For CV measurement of AuNCs, a 10 mM H_2_SO_4_ electrolyte was introduced into the channel, and a triangular potential sweep between 0 Vand 1.4 V with a scanning rate of 0.1 V/s was applied by an electrochemical workstation. For electrochemical dissolution of silver nanowires, a 1 M KSCN electrolyte was introduced into the channel, and a constant oxidizing potential of 1.5 V was applied.

During the cell experiments, square microfluidic channels with a side length of 10 mm and height of 1 mm were adopted (Supplementary Note S2), and an alternating current-modulated voltage of 4 V_pp_ was applied to the sensor surface with a certain frequency of 28 Hz. For the cell fixation, live cell imaging solution was first injected into the microfluidic channel to flow over the cells to obtain a baseline. Then, a 4% paraformaldehyde solution was introduced to chemically fix the cells. Finally, the flow was switched back to the live cell imaging solution to reach equilibration. The flow rate remained constant at 350 μL/min. For the cell stimulation, live cell imaging solution was first injected into the channel to flow over the cells to obtain a baseline. Then, 18 µM histamine in live cell imaging solution was introduced. The flow rate was kept constant at 300 μL/min.

## 3. Results

### 3.1. Optical Setup and Imaging Principles

**Figure 1:**
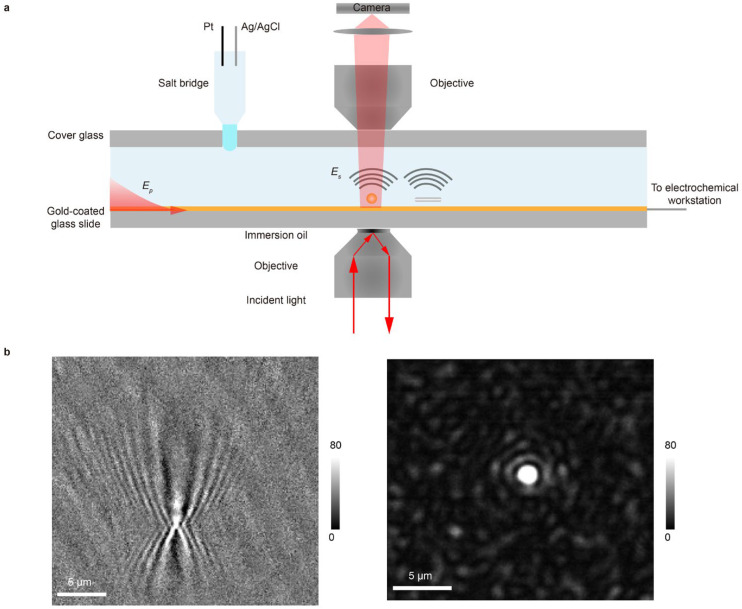
Setup and principle of EC-PSM. (**a**) Schematic diagram of the EC-PSM experimental setup. The gold-coated glass slide acts as both the imaging substrate and working electrode. Surface plasmon waves (*E_p_*) are excited by illuminating the gold film at the resonance angle through an oil-immersion objective, while a second objective above the sample collects plasmonic waves scattered directly from analytes (*E_s_*). The setup integrates Pt counter and Ag/AgCl reference electrodes into the microfluidic channel via an agarose gel salt bridge interface. (**b**) Point spread functions (PSFs) of nanoparticle imaging: (left panel) shows the PSF of SPR imaging and (right panel) shows the PSF of EC-PSM imaging.

The optical setup is based on a typical SPR imaging configuration. A gold-coated glass slide serves a dual purpose as both the imaging substrate and the working electrode. Surface plasmon waves are excited by illuminating the gold film through an oil-immersion objective at the resonance angle (Figure 1a). The reflected light is collected to form a conventional SPR image, in which contrast arises from the interference between the reflected planar plasmonic wave and the spherical waves scattered by surface-bound analytes. This generates a characteristic diffraction pattern: a central spot with a parabolic tail for each analyte, resulting in limited spatial resolution (Figure 1b). To achieve higher spatial resolution, we implement PSM. A second objective, placed above the sample, collects the plasmonic waves scattered directly from the analytes (Figure 1a). This arrangement bypasses the strong background reflection, producing high-contrast images and eliminating the parabolic diffraction tails. As a result, PSM delivers a markedly improved spatial resolution (Figure 1b, Supplementary Note S1).

Seamless integration with electrochemistry is essential for operando analysis in microfluidic environments. The gold film functions as the working electrode. To introduce the Pt counter and Ag/AgCl reference electrodes into the microfluidic channel without disturbing the optical path or fluid flow, we employ a salt bridge interface formed from an agarose gel. The gel forms a smooth, hydrophilic interface with the channel fluid, which helps to prevent bubble formation and stabilizes electrical contact (Figure 1a). Together, this integrated platform enables simultaneous high-resolution optical imaging and precise electrochemical modulation and interrogation within a single microfluidic device. To distinguish it from EC-SPR, we term this approach EC-PSM.

### 3.2. Detection of Nanoparticles

**Figure 2:**
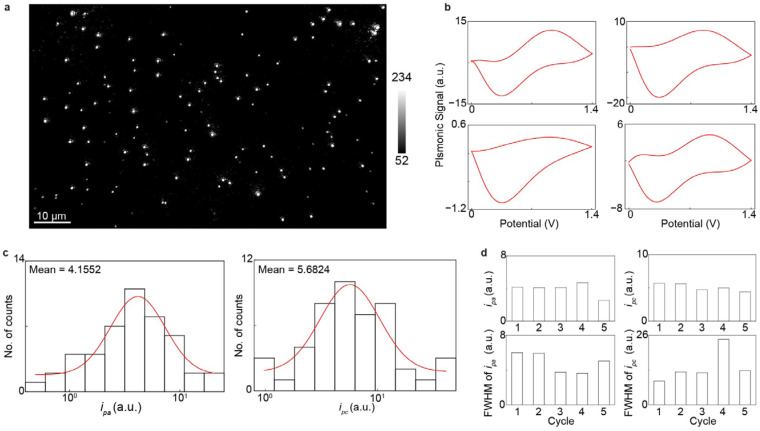
Imaging single gold nanocubes. (**a**) EC-PSM image of immobilized gold nanocubes. (**b**) Four representative cyclic voltammograms of individual gold nanocubes indicated in (**a**). (**c**) Histograms of oxidation peak current (*i_pa_*) and reduction peak current (*i_pc_*) of the entire 48 individual gold nanocubes within one cycle, where the red lines are achieved by Gaussian fitting. (**d**) Mean values and corresponding full-widths at half-maximum (FWHMs) of *i_pa_* and *i_pc_* histograms from entire nanocube population across five cycles.

To validate the EC-PSM platform, we first measured the CVs of AuNC. The nanocubes were immobilized on a dopamine-modified sensor surface. We then introduced a 10 mM H_2_SO_4_ solution into the microfluidic channels to serve as the electrolyte, and an electrochemical workstation applied a triangular potential sweep to perform the CV measurements. Figure 2a presents an EC-PSM image of the immobilized AuNCs, in which individual particles are clearly resolved due to the high spatial resolution of PSM. By tracking the image intensity changes with the applied triangular potential sweeping and performing deconvolution (Methods), we obtained the CVs for all individual AuNCs. Four representative CVs from different AuNCs are displayed in Figure 2b, revealing clear variations between particles. This individual heterogeneity is further illustrated by histograms of the oxidation (*i_pa_*) and reduction (*i_pc_*) peak currents for the entire population at the first potential sweeping cycle (Figure 2c). The shifts in peak positions and the full-widths at half-maximum (FWHMs) across different potential sweeping cycles indicate that the electrochemical activity and heterogeneity of the nanocubes evolve with repeated potential sweeping (Figure 2d). The individual heterogeneity of electrochemical activity may be due to non-uniform stabilizing ligands (CTAB) adsorption, (100) facet integrity/defect variation, and (100)-to-polycrystalline structural reconstruction rate variation during CV [28].

### 3.3. Detection of Nanowires

**Figure 3:**
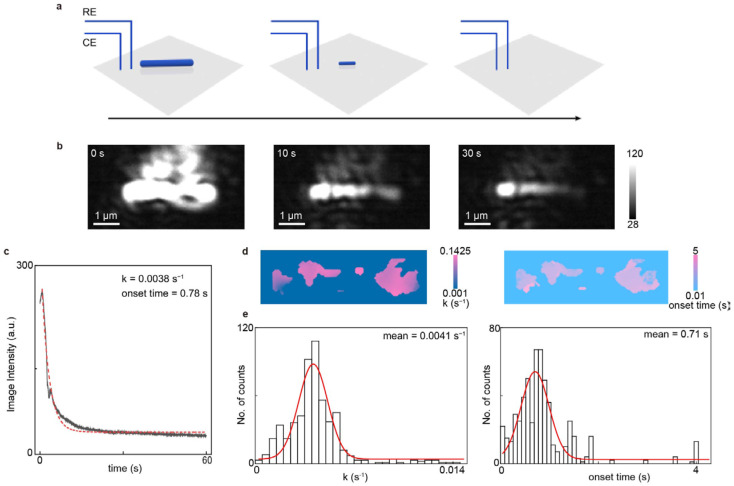
Imaging single nanowire. (**a**) Schematic diagram of the real-time process of electrochemical dissolution of an immobilized single silver nanowire, where the black arrow indicates the progression of time. (**b**) Representative EC-PSM image snapshots of the silver nanowire at 0 s, 10 s, and 30 s after applying a constant oxidizing potential. (**c**) Dissolution curve of the single silver nanowire, constructed by tracking the time-dependent changes in image intensity, averaged over the entire nanowire, where the red dotted line is achieved by exponential fitting. (**d**) Spatial distribution maps of the local dissolution rate and onset time of dissolution across the entire silver nanowire. (**e**) Histograms of the dissolution rates and onset times for all measured sites on the nanowire, where the red lines are achieved by Gaussian fitting. Three individual nanowires were analyzed.

To demonstrate the microscopic imaging and analysis capabilities of EC-PSM, we immobilized a single silver nanowire on the dopamine-modified sensor surface and monitored its electrochemical dissolution process (Figure 3 presents representative data and Supplementary Figure S1 provides more results). As shown in Figure 3b, the nanowire is clearly resolved, highlighting the superior spatial resolution of the PSM optical path. Subsequently, we applied a constant oxidizing potential to the system to induce the electrochemical dissolution of the silver nanowire under potentiation control. This process was monitored in real-time via EC-PSM, with representative frames shown in Figure 3b. By tracking the time-dependent changes in image intensity, averaged over the whole nanowire, we constructed a dissolution curve, revealing the kinetics of the reaction (Figure 3c). Initial observation of the dissolution sequence indicated significant heterogeneity along the nanowire, as shown in Figure 3b. To quantify this spatial variation, we mapped two key parameters across the nanowire’s structure, including the onset time of dissolution and the local dissolution rate (Figure 3d). This spatial mapping visually confirms that the electrochemical activity is not uniform. Further statistical analysis, presented in the histograms of the onset times and dissolution rates for all measured sites (Figure 3e), definitively demonstrates the pronounced spatial heterogeneity in the nanowire’s electrochemical behavior, which possibly arose from the spatial inhomogeneities of silver nanowire, including lengthwise structural variations (e.g., crystallographic orientation, grain boundaries and diameter), non-uniform stabilizing ligands adsorption, uneven electrode contact (tight/loose), and so on [29,30].

### 3.4. Analysis of Cell Fixation

**Figure 4:**
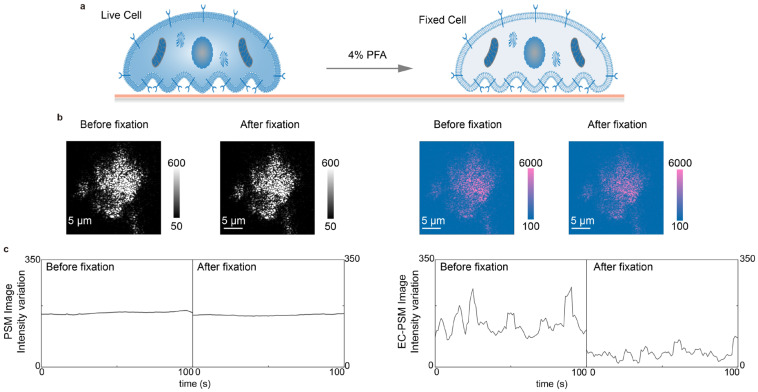
Analysis of cell fixation. (**a**) Schematic diagram of A549 cells cultured on the sensor surface before (left) and after (right) chemical fixation with 4% paraformaldehyde (PFA) solution in PBS. (**b**) Representative PSM images (left two panels) and EC-PSM images (right two panels) of A549 cells before and after fixation. (**c**) Image intensity changes in A549 cells before and after fixation under PSM mode (left panel) and EC-PSM mode (right panel). Two individual cells were analyzed.

In addition to performing CV, EC-PSM can also quantify surface charge density. This capability is based on the fact that PSM preserves the sharp plasmon resonance characteristic of conventional SPR. By switching the applied potential from a triangular sweep to a sinusoidal modulation, the amplitude of the resulting image intensity variation in the frequency domain corresponds directly to surface charge density, which is analogous to established EC-SPR methodology. To demonstrate this application, we cultured A549 cells directly on the sensor surface and subsequently introduced a 4% paraformaldehyde solution in PBS to chemically fix the cells (Figure 4a). Representative PSM and EC-PSM images acquired before and after fixation are shown in Figure 4b (Supplementary Figure S2 provides more results), indicating changes in the EC-PSM signal. By tracking the temporal evolution of image intensity during fixation, we obtained characteristic response curves reflecting cellular dynamics (Figure 4c). The intensity in the standard PSM channel showed little change throughout the process, indicating that the total cellular mass and adhesion footprint were largely unaffected by fixation. In contrast, the EC-PSM signal intensity decreased significantly, corresponding to a reduction in surface charge density. This result is consistent with the known biological effect of paraformaldehyde, which increases membrane permeability and likely facilitates the dissipation of the localized charge, thereby lowering the net charge density that is detectable at the cell interface.

### 3.5. Analysis of Cell Stimulation

**Figure 5:**
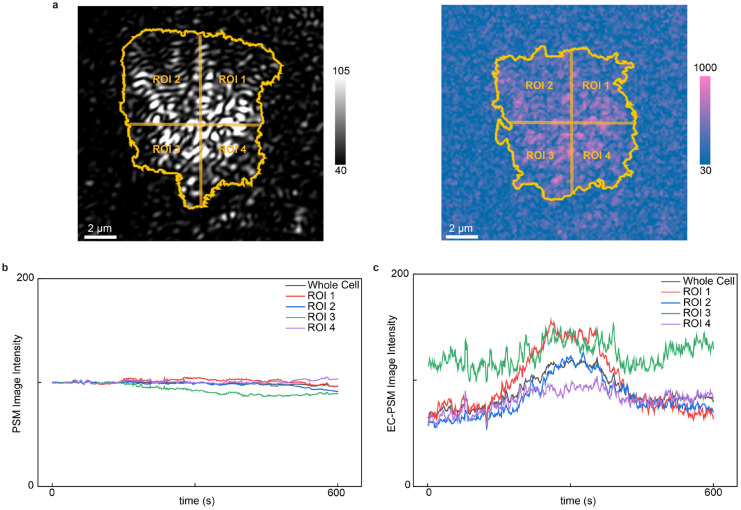
Analysis of cell stimulation. (**a**) Representative PSM image (**left**) and EC-PSM image (**right**) of HeLa cells with single cell adhesion region and four subcellular regions of interest (ROIs). (**b**) Dynamic changes in HeLa cell after histamine stimulation under PSM imaging mode. (**c**) Dynamic changes in HeLa cell after histamine stimulation under EC-PSM imaging mode. Two individual cells were analyzed.

Ion transport across the cellular membrane is a fundamental process regulating cellular activities. EC-PSM can analyze such dynamic ion fluxes in live cells by monitoring changes in local surface charge density. To demonstrate this capability, we cultured HeLa cells on the sensor surface and introduced histamine into the microfluidic channel (Figure 5 presents representative data and Supplementary Figure S3 provides more results). Histamine stimulates G-protein-coupled receptors (GPCRs), triggering an intracellular signaling cascade that results in calcium ion influx. PSM and EC-PSM images of HeLa cells are shown in Figure 5a. The high spatial resolution of both imaging modes enables subcellular analysis. Accordingly, we segmented individual cells into four distinct regions to investigate localized responses. By tracking the temporal intensity profiles in both the PSM and EC-PSM channels, we obtained the dynamic variations in cellular mass and surface charge density, respectively (Figure 5b,c). As anticipated, the PSM signal, which was reflective of cell mass, remained stable throughout the experiment, as histamine itself introduces negligible mass change. In contrast, the EC-PSM signal, corresponding to surface charge, exhibited a rapid initial increase followed by a swift decrease. This biphasic response is consistent with the expected physiology: histamine activation of GPCRs induces a rapid influx of positively charged calcium ions, which locally increases the surface charge density. Subsequently, compensatory ion movements and diffusion processes to re-establish osmotic and electrochemical equilibrium dissipate this localized charge, leading to the observed signal decay [31,32]. This experiment validates the utility of EC-PSM for real-time, high-resolution monitoring of ion transport dynamics during cellular stimulation.

## 4. Discussion

We introduce and validate EC-PSM, an integrated opto-electrochemical microfluidic platform that uniquely merges the microscopic spatial resolution of PSM with electrochemical control. By shifting from conventional reflection-based detection to a scattering-based scheme, EC-PSM overcomes two fundamental limitations of traditional EC-SPR: poor spatial resolution and high susceptibility to surface charging. The result is a synergistic tool that is capable of operando analysis, delivering spatially resolved maps of electrochemical activity and interfacial properties with single-entity sensitivity. The core innovation of EC-PSM lies in its direct detection of locally scattered plasmonic waves. In contrast to EC-SPR, where the signal arises from interference between propagating surface plasmon waves and analyte-induced scattering, producing long parabolic tails, EC-PSM directly collects the scattered light. This decouples the optical response from the long-range propagation of surface plasmon waves, effectively eliminating long parabolic tails and achieving a spatial resolution that is nearly an order of magnitude better than EC-SPR. This advantage is decisively demonstrated in experiments resolving individual nanoparticles and distinct electrochemically active sites along a single nanowire. Critically, EC-PSM enables the extraction of site-specific cyclic voltammograms and dissolution kinetics from single nano-objects, offering unprecedented access to electrocatalytic heterogeneity: a key frontier in nanoscience and energy research.

A further distinguishing feature of EC-PSM is its insensitivity to surface charging, a major source of noise in conventional EC-SPR. This robustness is essential for biological applications. In live-cell studies, EC-PSM clearly resolves quantifiable changes in interfacial charge density during chemical fixation and in response to GPCR-mediated calcium influx. The platform’s functionality hinges on a custom-designed microfluidic cell, integrating a salt bridge directly into the flow path. This architecture ensures stable, bubble-free potentiation control over microliter-scale sample volumes while preserving an unobstructed optical axis for high-resolution imaging. Such seamless co-integration of electrochemistry and optics is indispensable for probing dynamic processes in confined microenvironments, from nanoparticle dissolution under potential control to real-time cellular responses during continuous perfusion.

Despite its capabilities, EC-PSM has inherent constraints. It requires a plasmonic active substrate, and analytes must reside within the evanescent field of the sensor surface. Additionally, electrolyte conductivity must support both efficient electrochemical operation and plasmonic excitation. Future directions include expanding the range of compatible plasmonic substrates, incorporating complementary techniques such as electrochemical impedance spectroscopy, and extending the platform to multi-modal sensing. In summary, EC-PSM represents a significant advance in unified opto-electrochemical sensing. By combining nanoscale spatial resolution, electrochemical specificity, and microfluidic compatibility in a single platform, it opens new pathways for investigating heterogeneous electrochemical phenomena at the single-entity level and for performing real-time, label-free interrogation of complex biological interfaces. We anticipate that EC-PSM will become a powerful tool across disciplines, from fundamental electrochemistry and nanomaterials science to live-cell biophysics and point-of-care biosensing in complex media.

## Data Availability

The data presented in this study are available upon request from the corresponding author.

## Supplementary Materials

The following supporting information can be downloaded at: https://www.mdpi.com/article/10.3390/bios16020086/s1, Figure S1. Imaging single nanowire. (a) EC-PSM image snapshots of the silver nanowire at 0 s, 10 s, and 30 s after applying a constant oxidizing potential. (b) Dissolution curve of the single silver nanowire, constructed by tracking the time-dependent changes in image intensity averaged over the entire nanowire. (c) Spatial distribution maps of the local dissolution rate and onset time of dissolution across the entire silver nanowire. (d) Histograms of the dissolution rates and onset times for all measured sites on the nanowire. Figure S2. Analysis of cell fixation. (a) PSM images (left two panels) and EC-PSM images (right two panels) of A549 cells before and after fixation. (b) Image intensity changes of A549 cells before and after fixation under PSM mode (left panel) and EC-PSM mode (right panel). Figure S3. Analysis of cell stimulation. (a) PSM image (left) and EC-PSM image (right) of HeLa cells with single cell adhesion region and four subcellular regions of interest (ROIs). (b) Dynamic changes of HeLa cell after histamine stimulation under PSM imaging mode. (c) Dynamic changes of HeLa cell after histamine stimulation under EC-PSM imaging mode. Figure S4. Sensitivity of EC-SPR and EC-PSM to gold surface charging. Figure S5. Semi-open electrochemical microfluidic channel for gold nanocubes (AuNCs) and silver nanowires analysis. Figure S6. Electrochemical microfluidic channel for cell analysis.

## References

[1] Yager P., Edwards T., Fu E., Helton K., Nelson K., Tam M.R., Weigl B.H. (2006). Microfluidic diagnostic technologies for global public health. Nature.

[2] Pirone D., Lim J., Merola F., Miccio L., Mugnano M., Bianco V., Cimmino F., Visconte F., Montella A., Capasso M. (2022). Stain-free identification of cell nuclei using tomographic phase microscopy in flow cytometry. Nat. Photon..

[3] Aubry G., Lee H.J., Lu H. (2023). Advances in Microfluidics: Technical Innovations and Applications in Diagnostics and Therapeutics. Anal. Chem..

[4] Gebreyesus S.T., Muneer G., Huang C.C., Siyal A.A., Anand M., Chen Y.J., Tu H.L. (2023). Recent advances in microfluidics for single-cell functional proteomics. Lab Chip.

[5] Liao Z.R., Zhang Y., Li Y.R., Miao Y.F., Gao S.M., Lin F.K., Deng Y.L., Geng L.N. (2019). Microfluidic chip coupled with optical biosensors for simultaneous detection of multiple analytes: A review. Biosens. Bioelectron..

[6] Pour S.R.S., Calabria D., Emamiamin A., Lazzarini E., Pace A., Guardigli M., Zangheri M., Mirasoli M. (2023). Electrochemical vs. Optical Biosensors for Point-of-Care Applications: A Critical Review. Chemosensors.

[7] Liu X.-W., Yang Y., Wang W., Wang S., Gao M., Wu J., Tao N. (2017). Plasmonic-Based Electrochemical Impedance Imaging of Electrical Activities in Single Cells. Angew. Chem. Int. Ed..

[8] Wang W., Foley K., Shan X., Wang S., Eaton S., Nagaraj V.J., Wiktor P., Patel U., Tao N. (2011). Single cells and intracellular processes studied by a plasmonic-based electrochemical impedance microscopy. Nat. Chem..

[9] Wang S., Huang X., Shan X., Foley K.J., Tao N. (2010). Electrochemical Surface Plasmon Resonance: Basic Formalism and Experimental Validation. Anal. Chem..

[10] Shan X., Patel U., Wang S., Iglesias R., Tao N. (2010). Imaging Local Electrochemical Current via Surface Plasmon Resonance. Science.

[11] Shan X., Wang S., Wang W., Tao N. (2011). Plasmonic-Based Imaging of Local Square Wave Voltammetry. Anal. Chem..

[12] Lu J., Wang W., Wang S., Shan X., Li J., Tao N. (2012). Plasmonic-Based Electrochemical Impedance Spectroscopy: Application to Molecular Binding. Anal. Chem..

[13] MacGriff C., Wang S., Wiktor P., Wang W., Shan X., Tao N. (2013). Charge-Based Detection of Small Molecules by Plasmonic-Based Electrochemical Impedance Microscopy. Anal. Chem..

[14] Liang W., Wang S., Festa F., Wiktor P., Wang W., Magee M., LaBaer J., Tao N. (2014). Measurement of Small Molecule Binding Kinetics on a Protein Microarray by Plasmonic-Based Electrochemical Impedance Imaging. Anal. Chem..

[15] Wang Y., Shan X., Cui F., Li J., Wang S., Tao N. (2015). Electrochemical Reactions in Subfemtoliter-Droplets Studied with Plasmonics-Based Electrochemical Current Microscopy. Anal. Chem..

[16] Shan X., Chen S., Wang H., Chen Z., Guan Y., Wang Y., Wang S., Chen H.-Y., Tao N. (2015). Mapping Local Quantum Capacitance and Charged Impurities in Graphene via Plasmonic Impedance Imaging. Adv. Mater..

[17] Wang Y., Shan X., Wang S., Tao N., Blanchard P.-Y., Hu K., Mirkin M.V. (2016). Imaging Local Electric Field Distribution by Plasmonic Impedance Microscopy. Anal. Chem..

[18] Wang Y., Shan X., Wang H., Wang S., Tao N. (2017). Plasmonic Imaging of Surface Electrochemical Reactions of Single Gold Nanowires. J. Am. Chem. Soc..

[19] Yuan L., Tao N., Wang W. (2017). Plasmonic Imaging of Electrochemical Impedance. Annu. Rev. Anal. Chem..

[20] Wu G., Zhou X.L., Lv W.L., Qian C., Liu X.W. (2022). Real-Time Plasmonic Imaging of the Compositional Evolution of Single Nanoparticles in Electrochemical Reactions. Nano Lett..

[21] Zhang P., Ma G., Dong W., Wan Z., Wang S., Tao N. (2020). Plasmonic scattering imaging of single proteins and binding kinetics. Nat. Methods.

[22] Zhang P., Ma G., Wan Z., Wang S. (2021). Quantification of Single-Molecule Protein Binding Kinetics in Complex Media with Prism-Coupled Plasmonic Scattering Imaging. ACS Sens..

[23] Zhang P., Zhou X., Wang R., Jiang J., Wan Z., Wang S. (2021). Label-Free Imaging of Nanoscale Displacements and Free-Energy Profiles of Focal Adhesions with Plasmonic Scattering Microscopy. ACS Sens..

[24] Ma G., Zhang P., Zhou X., Wan Z., Wang S. (2022). Label-Free Single-Molecule Pulldown for the Detection of Released Cellular Protein Complexes. ACS Cent. Sci..

[25] Zhang P., Zhou X., Jiang J., Kolay J., Wang R., Ma G., Wan Z., Wang S. (2022). In Situ Analysis of Membrane-Protein Binding Kinetics and Cell–Surface Adhesion Using Plasmonic Scattering Microscopy. Angew. Chem. Int. Ed..

[26] Wu G., Lv W.-L., Qian C., Liu X.-W. (2024). High-throughput identification of single nanoparticles via electrochemically assisted high-resolution plasmonic scattering interferometric microscopy. Nano Lett..

[27] Ma K., Zhang Y., Liu L., Xi J., Qiu X., Guan T., He Y. (2019). In situ mapping of activity distribution and oxygen evolution reaction in vanadium flow batteries. Nat. Commun..

[28] Garcia A., Wang S., Tao N., Shan X., Wang Y. (2021). Plasmonic Imaging of Oxidation and Reduction of Single Gold Nanoparticles and Their Surface Structural Dynamics. Acs Sens..

[29] Wu G., Qian C., Lv W.L., Zhao X., Liu X.W. (2023). Dynamic imaging of interfacial electrochemistry on single Ag nanowires by azimuth-modulated plasmonic scattering interferometry. Nat. Commun..

[30] Li J., Lin P., Wu L., Yue Y., Ma G. (2025). Deciphering Complex Electrochemical Reaction Dynamics and Interactions of Single Nanoentities via Evanescent Scattering Microscopy. Angew. Chem. Int. Ed..

[31] Lohse M.J., Nikolaev V.O., Hein P., Hoffmann C., Vilardaga J.-P., Bünemann M. (2008). Optical techniques to analyze real-time activation and signaling of G-protein-coupled receptors. Trends Pharmacol. Sci..

[32] Lu J., Li J.H. (2015). Label-Free Imaging of Dynamic and Transient Calcium Signaling in Single Cells. Angew. Chem. Int. Ed..

