# Supplementary material for: Electrochemically Modulated Optical Imaging Sensors Integrated with Microfluidics
Source: Biosensors (Basel). 2026 Jan 30;16(2):86. doi: 10.3390/bios16020086 (PMC12938376; doi:10.3390/bios16020086)
Supplement: Supplementary file 1 [file biosensors-16-00086-s001.zip › biosensors-4093478-supplementary.pdf]

Supplementary Information

Electrochemically Modulated Optical Imaging Sensors Integrated with Microfluidics

Zehao Ye [1,2], Jiying Xu [1,2], Yi Chen [1,2] and Pengfei Zhang [1,2,*]

[1] Beijing National Laboratory for Molecular Sciences, Key Laboratory of Analytical Chemistry for Living Biosystems, Institute of Chemistry, Chinese Academy of Sciences, Beijing 100190, China;
yezh123@iccas.ac.cn (Z.Y.); xujy@iccas.ac.cn (J.X.); chenyi@iccas.ac.cn (Y.C.)
[2] University of Chinese Academy of Sciences, Beijing 100049, China
* Correspondence: pfzhang@iccas.ac.cn

Supplementary Figure S1

Imaging single nanowire

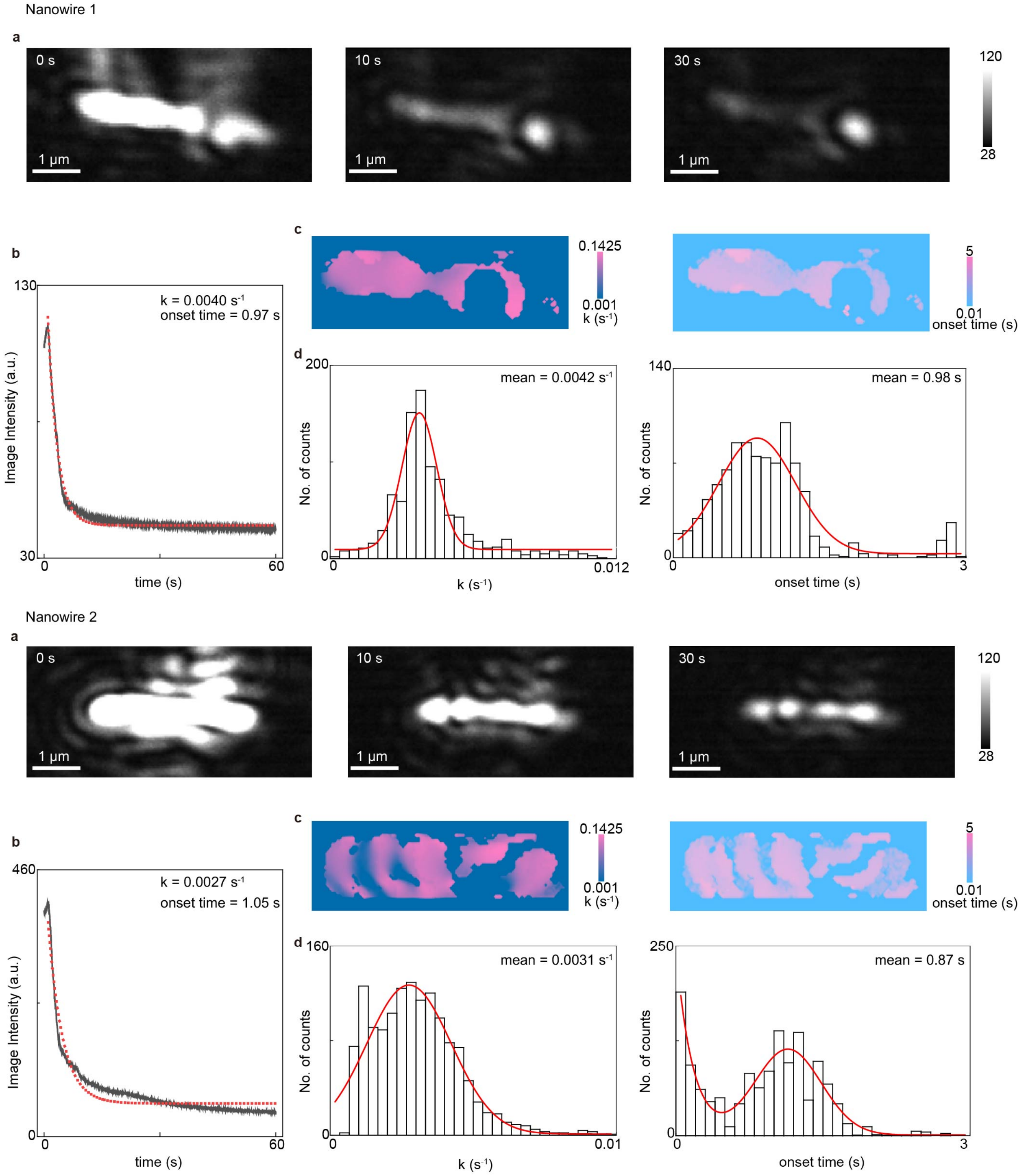

Figure S1. Imaging single nanowire. (a) EC-PSM image snapshots of the silver nanowire at 0 s, 10 s, and 30 s after applying a constant oxidizing potential. (b) Dissolution curve of the single silver nanowire,

constructed by tracking the time-dependent changes in image intensity averaged over the entire nanowire. (c) Spatial distribution maps of the local dissolution rate and onset time of dissolution across the entire silver nanowire. (d) Histograms of the dissolution rates and onset times for all measured sites on the nanowire.

Supplementary Figure S2

Analysis of cell fixation

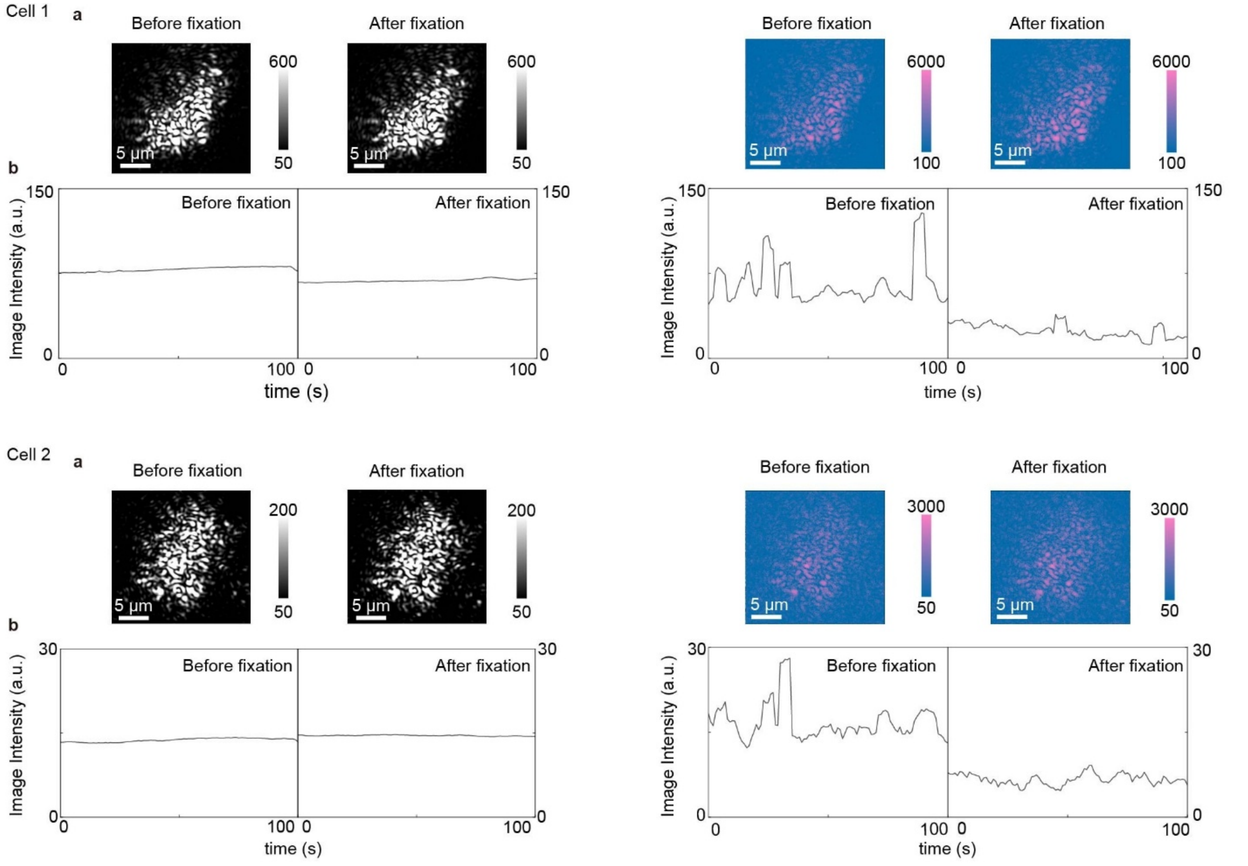

Figure S2. Analysis of cell fixation. (a) PSM images (left two panels) and EC-PSM images (right two panels) of A549 cells before and after fixation. (b) Image intensity changes of A549 cells before and after fixation under PSM mode (left panel) and EC-PSM mode (right panel).

Supplementary Figure S3

Analysis of cell stimulation

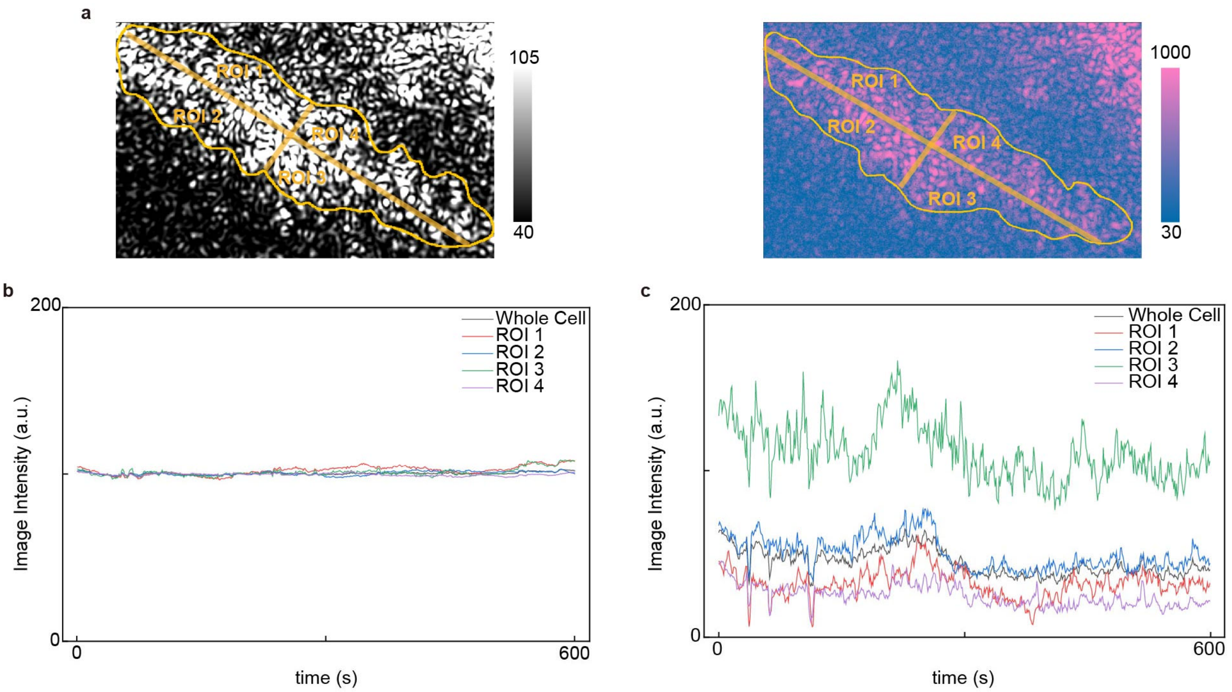

Figure S3. Analysis of cell stimulation. (a) PSM image (left) and EC-PSM image (right) of HeLa cells with single cell adhesion region and four subcellular regions of interest (ROIs). (b) Dynamic changes of HeLa cell after histamine stimulation under PSM imaging mode. (c) Dynamic changes of HeLa cell after histamine stimulation under EC-PSM imaging mode.

Supplementary Note S1

Comparison between EC-PSM and EC-SPR

1. Higher resolution of EC-PSM

EC-PSM has higher resolution than EC-SPR, which can be obtained from their imaging principles (Nat Methods. 2020,17(10): 1010-1017).

In EC-SPR, light reflected from the gold surface is collected to form an SPR image described as

$$I \sim \left|E_p + E_s + E_r\right|^2 \quad \text{(N1.1)}$$

where E_p is the excited plasmonic wave, E_s is the scattering of the plasmonic wave by an analyte on the sensor surface, and E_r is the reflection of the incident wave from the backside of the gold surface. The interference between the planar plasmonic wave and the spherical scattered plasmonic wave is given by $2\left|E_p\right|\left|E_s\right|cos(\theta)$, where θ is the phase difference beween the two waves, which determines the SPR image contrast and produces a spot at the location of the analyte with a parabolic tail. E_r produces a large background in the SPR image.

While in EC-PSM, plasmonic waves scattered by the analyte are collected by an objective placed on top of the sample to form a PSM image described as

$$I \sim |E_b + E_s|^2 = |E_b|^2 + 2|E_b||E_s|\cos\beta + |E_s|^2 \quad \text{(N1.2)}$$

where E_b is the scattering of the surface plasmonic waves scattered by the inherent sensor surface roughness, β is the phase difference between the light sattered by the analyte and by the sensor surface. The PSM image eliminates the parabolic tail, and avoid the collection of the strong reflection, providing a high contrast image. As a result, PSM delivers markedly improved spatial resolution.

2. Insensitivity of EC-PSM to surface charging

In EC-SPR, gold surface charging can cause the change of SPR condition, which will result into high background. While in EC-PSM, when the applid potential is not enough to cause electrochemical reaction on the gold surface, PSM demonstrates remarkable insensitivity to surface charging, effectively isolating the optical response to specific faradaic events and drastically improving the signal-to-background ratio (Supplementary Figure S4).

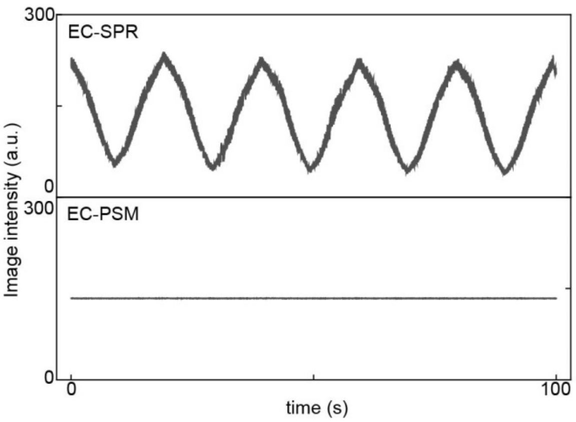

Figure S4. Sensitivity of EC-SPR and EC-PSM to gold surface charging.

Supplementary Note S2

Electrochemical microfluidic channel

For gold nanocubes (AuNCs) and silver nanowires analysis, a semi-open square microfluidic channel with a side length of 10 mm and height of 1 mm were constructed by a bottom gold chip, double-sided tape spacers, and an upper cover glass as shown in Supplementary Figure S5. One hole in the upper cover glass is used to employ a salt bridge to introduce Pt counter and Ag/AgCl reference electrodes into the channel. The solution is added in the channel from one open side with a pipette and removed from the other open side with absorbing paper.

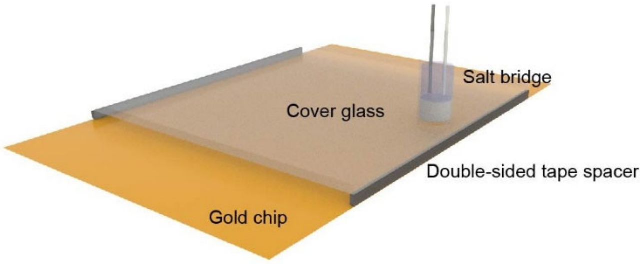

Figure S5. Semi-open electrochemical microfluidic channel for gold nanocubes (AuNCs) and silver nanowires analysis.

For cell analysis, a square microfluidic channel with a side length of 10 mm and height of 1 mm were constructed by a bottom gold chip, double-sided tape spacers, and an upper cover glass as shown in Supplementary Figure S6. There are three holes in the upper cover glass, one used to employ a salt bridge to introduce Pt counter and Ag/AgCl reference electrodes into the channel and the other two used to the inflow and outflow of fluids.

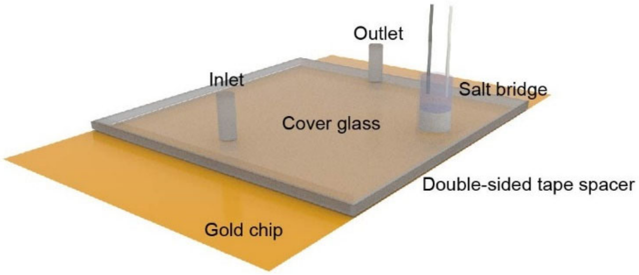

Figure S6. Electrochemical microfluidic channel for cell analysis.
